# Comparative analysis of macroalgae supplementation on the rumen microbial community: *Asparagopsis taxiformis* inhibits major ruminal methanogenic, fibrolytic, and volatile fatty acid-producing microbes *in vitro*

**DOI:** 10.3389/fmicb.2023.1104667

**Published:** 2023-04-03

**Authors:** Eóin O’Hara, Stephanie A. Terry, Paul Moote, Karen A. Beauchemin, Tim A. McAllister, D. Wade Abbott, Robert J. Gruninger

**Affiliations:** Lethbridge Research and Development Centre, Agriculture and Agri-Food Canada, Lethbridge, AB, Canada

**Keywords:** methane, rumen, *Asparagopsis*, seaweed, livestock, greenhouse gas, microbiome

## Abstract

Seaweeds have received a great deal of attention recently for their potential as methane-suppressing feed additives in ruminants. To date, *Asparagopsis taxiformis* has proven a potent enteric methane inhibitor, but it is a priority to identify local seaweed varieties that hold similar properties. It is essential that any methane inhibitor does not compromise the function of the rumen microbiome. In this study, we conducted an *in vitro* experiment using the RUSITEC system to evaluate the impact of three red seaweeds, *A. taxiformis, Palmaria mollis,* and *Mazzaella japonica*, on rumen prokaryotic communities. 16S rRNA sequencing showed that *A. taxiformis* had a profound effect on the microbiome, particularly on methanogens. Weighted Unifrac distances showed significant separation of *A. taxiformis* samples from the control and other seaweeds (*p* < 0.05). Neither *P. mollis* nor *M. japonica* had a substantial effect on the microbiome (*p* > 0.05). *A. taxiformis* reduced the abundance of all major archaeal species (*p* < 0.05), leading to an almost total disappearance of the methanogens. Prominent fiber-degrading and volatile fatty acid (VFA)-producing bacteria including *Fibrobacter* and *Ruminococcus* were also inhibited by *A. taxiformis* (*p* < 0.05), as were other genera involved in propionate production. The relative abundance of several other bacteria including *Prevotella*, *Bifidobacterium, Succinivibrio, Ruminobacter*, and unclassified *Lachnospiraceae* were increased by *A. taxiformis* suggesting that the rumen microbiome adapted to an initial perturbation. Our study provides baseline knowledge of microbial dynamics in response to seaweed feeding over an extended period and suggests that feeding *A. taxiformis* to cattle to reduce methane may directly, or indirectly, inhibit important fiber-degrading and VFA-producing bacteria.

## 1. Introduction

To combat the ongoing climate change crisis, international legislative agreements have mandated limits on global warming to 1.5°C and 2°C by 2030 and 2050, respectively (Arndt et al., 2022), targets which are unlikely to be met if global food systems continue business as usual practices (Clark et al., 2020). Food production industries are significant sources of anthropogenic carbon, contributing around 30% of total greenhouse gas (GHG) emissions annually, with livestock responsible for ~30% of this total (Arndt et al., 2022). Methane (CH_4_) is produced as a normal by-product of microbial feed digestion in the rumen and is the principal source of carbon emissions from livestock (IPCC, 2018). CH_4_ has a 100-year global warming potential at least 28-times greater than that of CO_2_, and enteric methanogenesis contributes around 40% of all livestock GHG emissions (Gerber et al., 2013). In addition to the environmental impacts, ruminal methanogenesis reduces the dietary energy available to the animal by up to 12%; developing effective CH_4_ mitigation strategies is attractive from both environmental and economic perspectives (Johnson and Johnson, 1995).

Vaccination, selective breeding, dietary manipulation, and improvements in feed efficiency have all been examined for their efficacy in CH_4_ mitigation, with varying results (Gerber et al., 2013). The addition of certain seaweed species and their by-products to the diets of ruminant livestock has been extensively investigated and presents a promising and renewable approach to mitigate enteric CH_4_ emissions (Abbott et al., 2020). Several species of red and brown macroalgae are known to inhibit microbial methanogenesis when included as a feed additive in the diet of ruminants (Machado et al., 2014, 2016a, 2018; Roque et al., 2019). Among them, the bromoform containing red seaweed *Asparagopsis taxiformis* has been shown to have anti-methanogenic properties and can reduce CH_4_ production by over 95% *in vitro* and *in vivo* (Machado et al., 2016b; Roque et al., 2019, 2021; Terry et al., 2022). *A. taxiformis* is native to warm temperate waters and considered invasive elsewhere; therefore, the identification of local varieties of seaweeds that can favorably impact rumen function is a great priority. In addition to the potential role of seaweeds to reduce enteric CH_4_ emissions, there is also evidence that other bioactive compounds present in seaweeds may confer other benefits such as improved gut health and pathogen suppression (Belanche et al., 2016; Zhou et al., 2018).

Recently we examined the effects of three red seaweeds, *A. taxiformis, Mazzaella japonica*, and *Palmaria mollis*, on microbial fermentation and CH_4_ production in a simulated rumen environment over 21 days (Terry et al., 2022). In this experiment, rapid changes in gas production and fermentation parameters were observed during the first four days that seaweed was supplemented as the system adapted to the additive (days 8–11). This period was followed by an intermediate phase (days 12–16) in which small changes in the system were observed, and a stable phase (days 17–21) when the system had stabilized (Terry et al., 2022). The inclusion of *A. taxiformis*, resulted in an 84.0% reduction of CH_4_ production within the first 48 h, continuing to drop throughout the course of the experiment resulting in a 95.1% reduction in CH_4_ compared to controls during the stable phase (Terry et al., 2022). Accompanying the decrease in CH_4_ production, *A. taxiformis* significantly reduced acetate, propionate and total VFA production throughout the course of the experiment. *A. taxiformis* was found to reduce organic matter (OM) and neutral detergent fiber (NDF) digestibility compared to controls during the adaptation phase. Interestingly, digestibility recovered in the stable phase suggesting that adaptation to *A. taxiformis* occurred (Terry et al., 2022). In contrast, *M. japonica* and *P. mollis* did not have a measurable effect on *in vitro* fermentation or digestibility.

It is not clear how the inclusion of *A. taxiformis, M. japonica*, and *P. mollis* in diets influences the rumen microbiome and understanding of the microbial mechanisms underpinning ruminal adaptation to seaweed supplementation is generally lacking. To address these gaps, we employed 16S rRNA amplicon sequencing to assess the temporal dynamics of the rumen microbiome in response to supplementation with *A. taxiformis, M. japonica*, and *P. mollis* over a 13-day period using a RUSITEC apparatus. These data provide insight into rumen microbial adaptation to seaweed feeding, but also highlight potentially negative impacts on key rumen functions concurrent to CH_4_ inhibition.

## 2. Materials and methods

### 2.1. RUSITEC system

Details of seaweed procurement, RUSITEC design, sampling protocols have been published elsewhere (Terry et al., 2022). Donor cows used in this experiment were cared for in accordance with the guidelines of the Canadian Council on Animal Care (Canadian Council on Animal Care, 2009) under an approved animal ethics protocol ACC1830. This experiment was performed in duplicate using two identical RUSITEC apparatus equipped with eight fermentation vessels each. The experiment was divided into four phases: baseline (days 0–7), adaptation (days 8–11), intermediate (days 12–16) and stable (days 17–21). Each fermenter was inoculated with rumen fluid collected from 3 ruminally canulated beef heifers. The basal substrate (10 g dry matter (DM) 50:50 barley silage:barley straw) was added daily to the fermenters. The three seaweeds, *Asparagopsis taxiformis, M. japonica*, and *P. mollis* were added to the basal substrate on a 2% DM basis from day 8 onward. The control vessels were treated in an identical manner with no seaweed added. Beginning on day 8, A 1.5 mL sample of digester fluid was collected daily from each vessel for microbial community analysis. The sample was centrifuged at 21,000 RCF for 5 min to pellet the microbial cells. The supernatant was discarded, and the cell pellet was re-suspended in DNA/RNA Shield (Zymo Research), snap frozen in liquid nitrogen and stored at −80°C, pending molecular analysis.

### 2.2. Bromoform content analysis

The concentration of bromoform in each of the three seaweeds was determined using a Shimadzu QP2010 Ultra GC/MS system at a commercial laboratory (Bigelow Analytical Services, United States). Bromoform concentration was recorded as mg/g dry weight.

### 2.3. DNA isolation

Microbial DNA was isolated from all samples using the ZymoBIOMICS DNA Miniprep Kit, following the manufacturer’s instructions with some modifications; the cell pellet was re-suspended in 1,000 μL of DNA/RNA Shield (Zymo Research) and 250 μL of this cell suspension was added to 250 μL of lysis buffer in ZR BashingBead™ lysis tubes (Zymo Research) containing 0.1 mm and 0.5 mm beads. All samples underwent 5 rounds of bead beating using a MP FastPrep 24 (MP Bio, United States) for 20 s at 6.0 m/s with 1 min breaks between rounds. Following bead beating, samples were incubated at 70°C for 10 min to increase the effectiveness of DNA isolation. The remaining purification steps followed the manufacturers protocol, and all samples were eluted in 100 μL of distilled H_2_O. DNA quantity and quality was assessed with a Nanodrop 2,100 spectrophotometer and visualization in a 1% agarose gel.

### 2.4. PCR amplification, library construction, and sequencing

Sequencing was performed at the Genome Quebec Innovation Center (Montreal, Canada) using the Illumina MiSeq Reagent Kit v2 (500 cycle) following the manufacturer’s guidelines. The primers 515F (5′-GTGCCAGCMGCCGCGGTAA-3′) and 806R (5′-GGACTACHVGGGTWTCTAAT-3′) targeting the V4 region of the 16S rRNA gene were used to examine both bacterial and archaeal diversity (Caporaso et al., 2012). A 33 cycle PCR using 1 μl of a 1 in 10 dilution of genomic DNA and the Fast Start High Fidelity PCR System (Roche, Montreal, PQ) was conducted with the following conditions: 94°C for 2 min, followed by 33 cycles of 94°C for 30 s, 58°C for 30 s, and 72°C for 30 s, with a final elongation step at 72°C for 7 min. Fluidigm Corporation (San Francisco, CA) barcodes were incorporated in a second PCR reaction using the following conditions: 95°C for 10 min, followed by 15 cycles of 95°C for 15 s, 60°C for 30 s, and 72°C for 1 min, followed by a final elongation step at 72°C for 3 min. After amplification, PCR products were assessed in a 2% agarose gel to confirm adequate amplification. All samples were quantified using the Quant-iT PicoGreen dsDNA Assay Kit (Life Technologies, Carlsbad, CA) and were pooled in equal proportions. Pooled samples were then purified using calibrated Ampure XP beads (Beckman Coulter, Mississauga, ON). The pooled samples (library) were quantified using the Quant-iT PicoGreen dsDNA Assay Kit (Life Technologies, Carlsbad, CA) and the Kapa Illumina GA with Revised Primers-SYBR Fast Universal kit (Kapa Biosystems, Wilmington, MA). Average fragment size was determined using a LabChip GX (PerkinElmer, Waltham, MA, United States) instrument.

### 2.5. Sequence data analysis

Raw reads were imported in .fastq format to a local server for analysis. Read quality was evaluated using FASTQC (Andrews, 2010) and MultiQC (Ewels et al., 2016). Data were processed using the QIIME2 software package (Bolyen et al., 2019). FIGARO (Sasada et al., 2020) was used to identify optimum truncation positions for read merging. Reads were denoised into amplicon sequencing variants (ASVs) using the DADA2 (Callahan et al., 2016) plugin for QIIME2. A phylogenetic tree was generated using MAFFT (Katoh and Standley, 2013). ASVs were taxonomically classified using a Native Bayes classifier trained on the V4 region of the 16S rRNA gene using the Sci-Kit plugin in QIIME2. The SILVA SSU (v.138.1) (Quast et al., 2013) database was used to classify bacterial sequences while the Rumen and Intestinal Methanogens (RIM) database (v.14.7) (Seedorf et al., 2014) was used for archaeal reads. Initial analysis (described in Supplementary Figures S3A–C) indicated that increasing the confidence threshold to 0.85 (from a default of 0.7) prevented spurious ASV classification using the RIM database.

### 2.6. Diversity and correlation analysis

QIIME2 objects (ASV frequency table, taxonomy table, and phylogenetic tree) were imported into R as a Phyloseq (McMurdie and Holmes, 2013) object using the qiime2R package (Bisanz, 2018). Analysis was performed separately for bacterial and archaeal datasets. In-house R scripts were used to calculate summary statistics of read counts and distributions. ASVs that were not assigned to at least a microbial phylum were discarded. Rarefaction curves were generated to identify suitable subsampling thresholds for diversity analyses. Rarified data was used for ɑ- (Chao1 and Shannon) and β-diversity (Weighted Unifrac) calculations. ɑ-Diversity data was summarized according to phase for comparison using a two-way analysis of variance (ANOVA) with Tukeys post-hoc test. Statistically significant differences were declared at Bonferroni-adjusted *p* < 0.05. Permutational Analysis of Variance (PERMANOVA) tests were performed using the Vegan (Oksanen et al., 2022) R package, with pairwise tests performed using the RVaideMomoire R package (Herve, 2021). Homogeneity of dispersion (β-dispersion) tests were performed using Vegan. Canonical correspondence analysis (CCA) between rarified ASV count data and environmental data collected from the RUSITEC system was also performed using Vegan. Statistically significant differences were declared at a threshold of *p* < 0.05, and all figures were generated using ggplot2 in R (Wickham, 2009). Core rumen taxa were declared as those present in more than 50% of the samples and represented by at least 100 sequencing reads. Correlation analysis was performed between selected fermentation metrics and (i) the core microbiome and (ii) differentially abundant taxa using log10 transformed count data and Spearman correlations in R.

### 2.7. Differential abundance testing

Differentially abundant (DA) bacterial taxa between seaweed and control samples were identified using ANCOM-BC (Lin and Peddada, 2020) and ALDEx2 (Fernandes et al., 2014). Sequence data were arranged according to experimental phase to identify DA taxa between seaweed treatments within the adaptation (days 8–11); intermediate (days 12–16) and stable (days 17–21) phases. Prior to DA testing, low-prevalence features - those present in <10% of the samples – were discarded as recommended for microbiome data (Nearing et al., 2022). Non-rarefied data were used as input for all DA tests. For ANCOM-BC, the phyloseq object was passed to the ‘ancombc’ function and run at a maximum of 1,000 iterations. Structural zeros - taxa present in one group but absent in another – were declared as DA where present. A pseudocount of 1 was added to all observations to facilitate log transformation. Significant features were those with a Benjamini-Hochberg-adjusted *p* < 0.05. For ALDEx2, count data and metadata were passed to the ‘aldex’ command which employs a Dirichlet-multinomial model to infer abundance from counts. *p*-values generated from a Wilcoxon Rank Sum test were FDR-corrected using the Benjamini-Hochberg procedure and significant differences according to treatment group were declared at corrected *p* < 0.05. The significantly different taxa identified by both tools were compared and consensus taxa (i.e., those identified using both methods) declared as DA for each respective comparison.

## 3. Results

### 3.1. Bromoform analysis

Only *A. taxiformis* had detectable amounts of bromoform, at 0.517 mg/g^−1^ DM. Any bromoform present in *P. mollis* and *M. japonica* was below the limit of detection for the instrument.

### 3.2. Sequencing data characteristics

**Figure 1:**
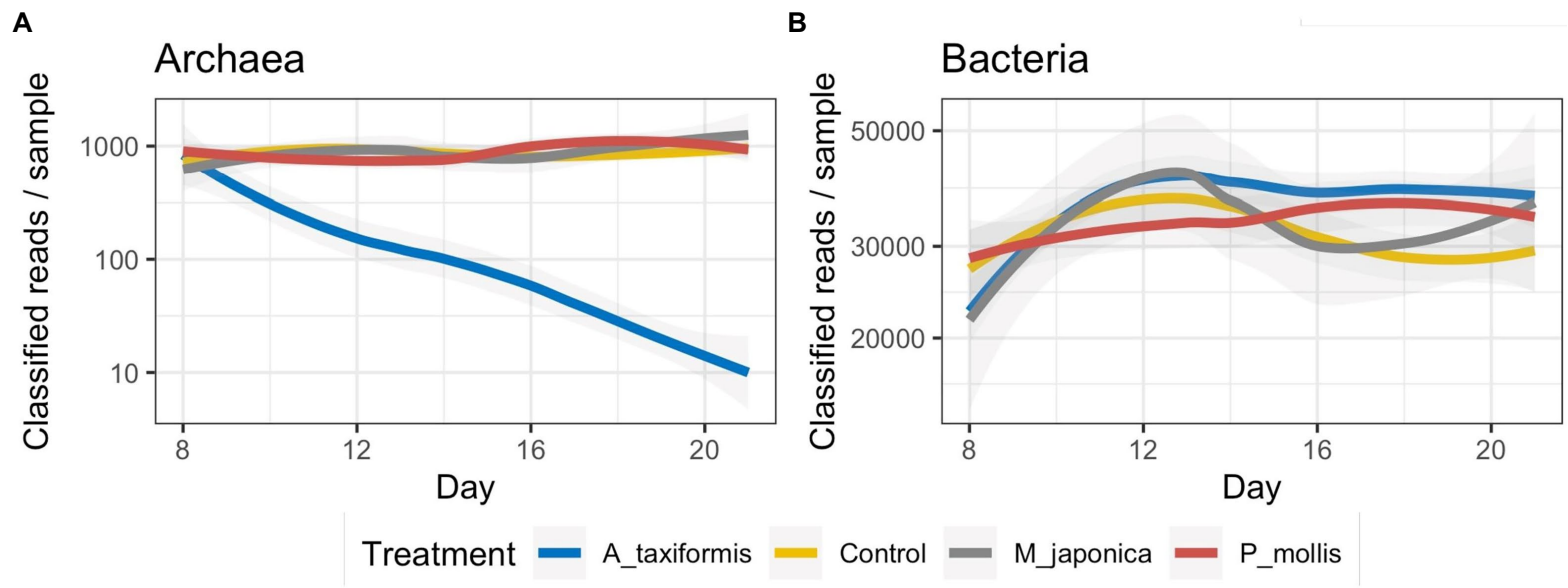
Number of reads denoised into **(A)** archaeal and **(B)** bacterial ASVs. Archaeal data is presented on a logarithmic scale for clarity.

Sequencing of 16S rRNA V4 amplicons from 272 digesta samples generated a total of 12,456,461 reads (range: 2,663–78,773) with an average of 45,795 ± 10,306 (mean ± SD) per sample. Denoising with DADA2 retained 72.6% of the reads and identified a total of 18,925 ASVs. Rarefaction curves for both bacterial and archaeal annotation data reached a plateau (Supplementary Figures S1A,B) indicating that our sampling depth was sufficient. When the total number of reads successfully denoised into archaeal ASVs were plotted over time, there was a clear temporal decline of archaea in the *A. taxiformis* samples with only 30 archaeal reads successfully denoised on D21 among the four samples (Figure 1A). A similar trend was not evident for the other seaweed treatments. The number of successfully denoised bacterial reads did not appear to differ dramatically across treatments throughout the experimental period though there were some temporal patterns evident (Figure 1B).

### 3.3. Microbial data analysis

#### 3.3.1. Alpha and beta diversity

**Figure 2:**
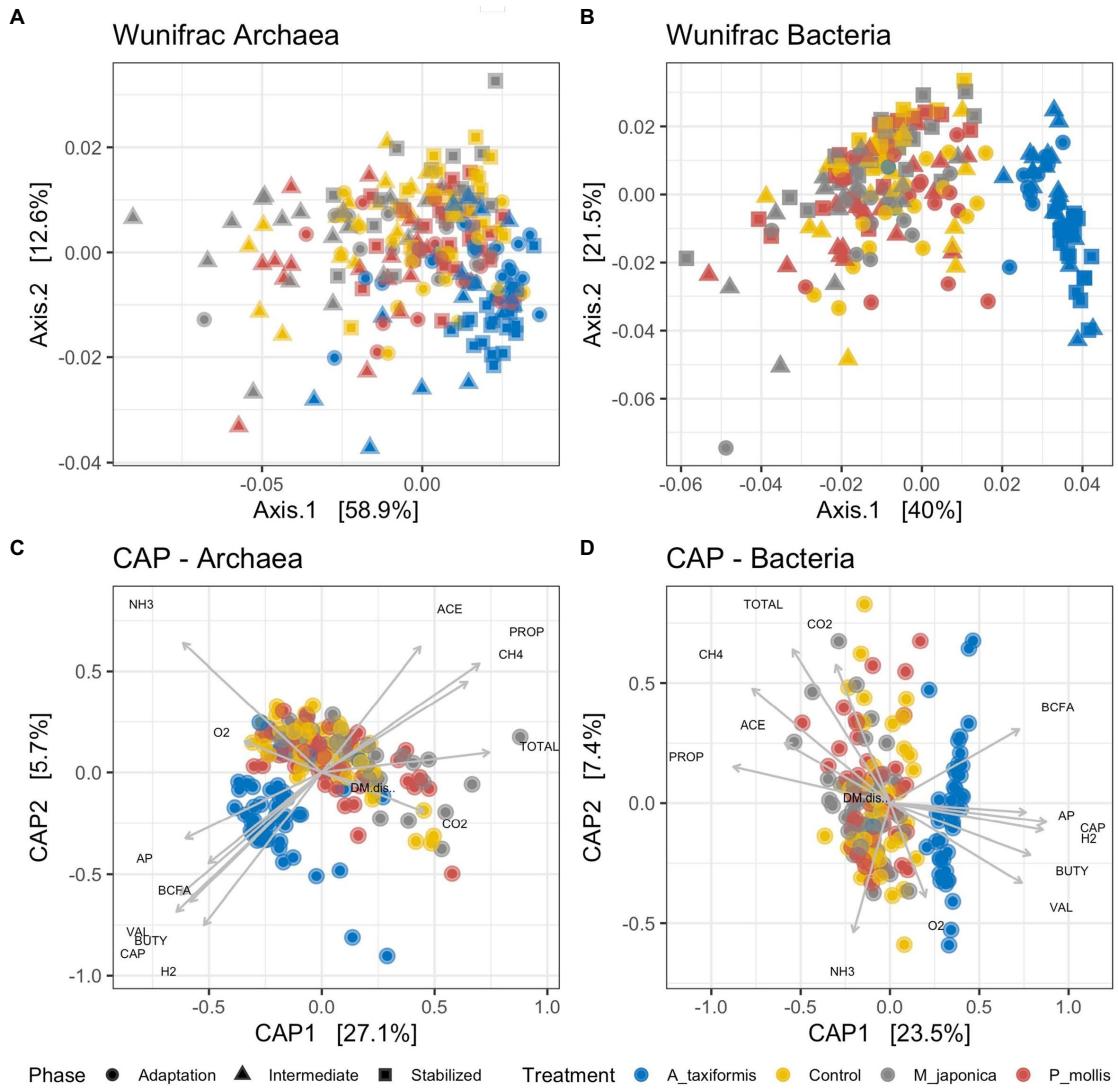
Unconstrained principal coordinate analysis (PCoA) plots based on rarefied Weighted Unifrac distance matrices for **(A)** archaeal and **(B)** bacterial communities. Constrained canonical analysis of principal coordinates (CAP) plots for **(C)** archaeal and **(D)** bacterial communities and environmental variables. The vectors indicate the axis that is best explained by each variable. Data was generated using Weighted Unifrac distance matrices. ACE, acetate; PROP, propionate; CH4, methane; TOTAL, total VFA; CO2, carbon dioxide; BCFA, branched-chain fatty acids; AP, acetate: propionate ratio; CAP, Caproate; H2, Hydrogen; BUTY, butyrate; VAL, valerate; O2, oxygen; NH3, ammonia. Individual sample values for all measurements are provided in the Supplementary material.

Principal coordinate (Figures 2A,B) and PERMANOVA (Table 1) analyses based on weighted Unifrac distances showed separation of both bacterial and archaeal microbial profiles according to treatment and phase (*p* < 0.05), with the *A. taxiformis* samples clearly separating from the other groups (Figures 2A,B). The separation increased throughout the experiment and was greatest in the stable phase. The *R*^2^ value was greater for treatment than experimental phase (0.30 vs. 0.04) in the bacterial data, indicating this was the largest factor contributing to compositional differences. Experimental phase and treatment made similar (0.17 vs. 0.18) contributions to dissimilarity of the archaeal data. Canonical correspondence analysis (Figures 2C,D) using the fermentation data reported by Terry et al. (2022) showed that the separation of the *A. taxiformis* samples from the other groups could be explained by several environmental measurements including: the decline in CH_4_, the concordant increase in H_2_, and the lower molar proportions of propionate and acetate (Terry et al., 2022). The environmental parameters (Supplementary Table S1) that drove the separation of samples was similar for both the bacterial and archaeal datasets (Figures 2C,D).

**Table 1:** Results of PERMANOVA analysis on bacterial and archaeal community dissimilarities.

Variable	*R^2^	^†^*F*-value	*p*-value	*R* ^2^	*F*-value	*p*-value
	Bacteria			Archaea		
Treatment	0.30	35.63	0.001	0.18	20.02	0.001
Phase	0.05	8.41	0.001	0.17	28.40	0.001
Day	0.01	2.18	0.065	0.01	2.60	0.064
Treatment:Day	0.07	7.75	0.001	0.02	2.25	0.025
Treatment:Phase	0.02	1.03	0.416	0.03	1.44	0.124
Residual	0.56	–	–	0.60	–	–
Total	1.00	–	–	1.00	–	–

Tests conducted using a weighted Unifrac dissimilarity matrix. ^*^R2: Proportion of variance explained by each variable; ^^†^^F-value, estimate of effect size.

**Figure 3:**
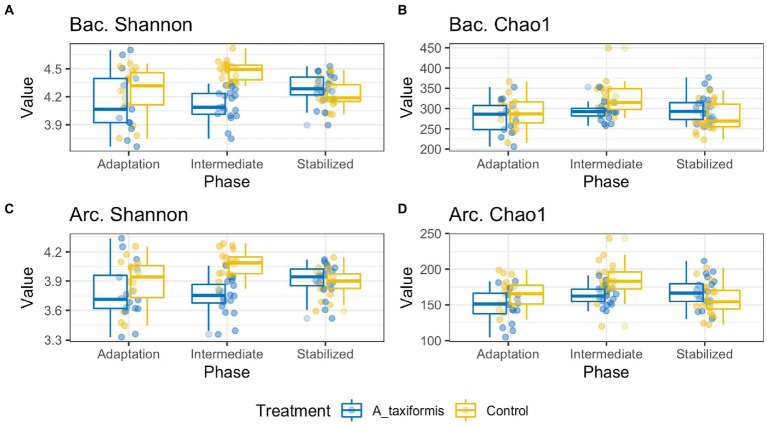
Alpha diversity boxplots of bacterial (Bac) and archaeal (Arc) sequencing data. Samples are grouped according to phase. All data was rarefied to an even depth prior to metric calculation. Panels **(A–D)** depict Shannon Evenness and Chao1 Richness metrics for *A. taxiformis* and control samples.

ANOVA analysis indicated that Chao1 and Shannon indices were influenced by treatment (*p* < 0.05), phase (*p* < 0.05), and the interaction of the two (*p* < 0.05). Only *A. taxiformis* exhibited major differences in alpha-diversity when compared to the controls (Figure 3), while differences between the controls and the other two seaweeds were minor. Results of all alpha-diversity comparisons are presented in Supplementary Tables S2, S3. *A. taxiformis* samples had lower Chao1 and Shannon index values in the intermediate phase compared to the controls (*p* < 0.05), while the *P. mollis, M. japonica* and Control samples had similar values. Treatment-wise differences declined for all treatments by the stable phase with few statistically significant differences evident (Supplementary Tables S2, S3).

#### 3.3.2. Microbial community composition and response to seaweed addition

**Figure 4:**
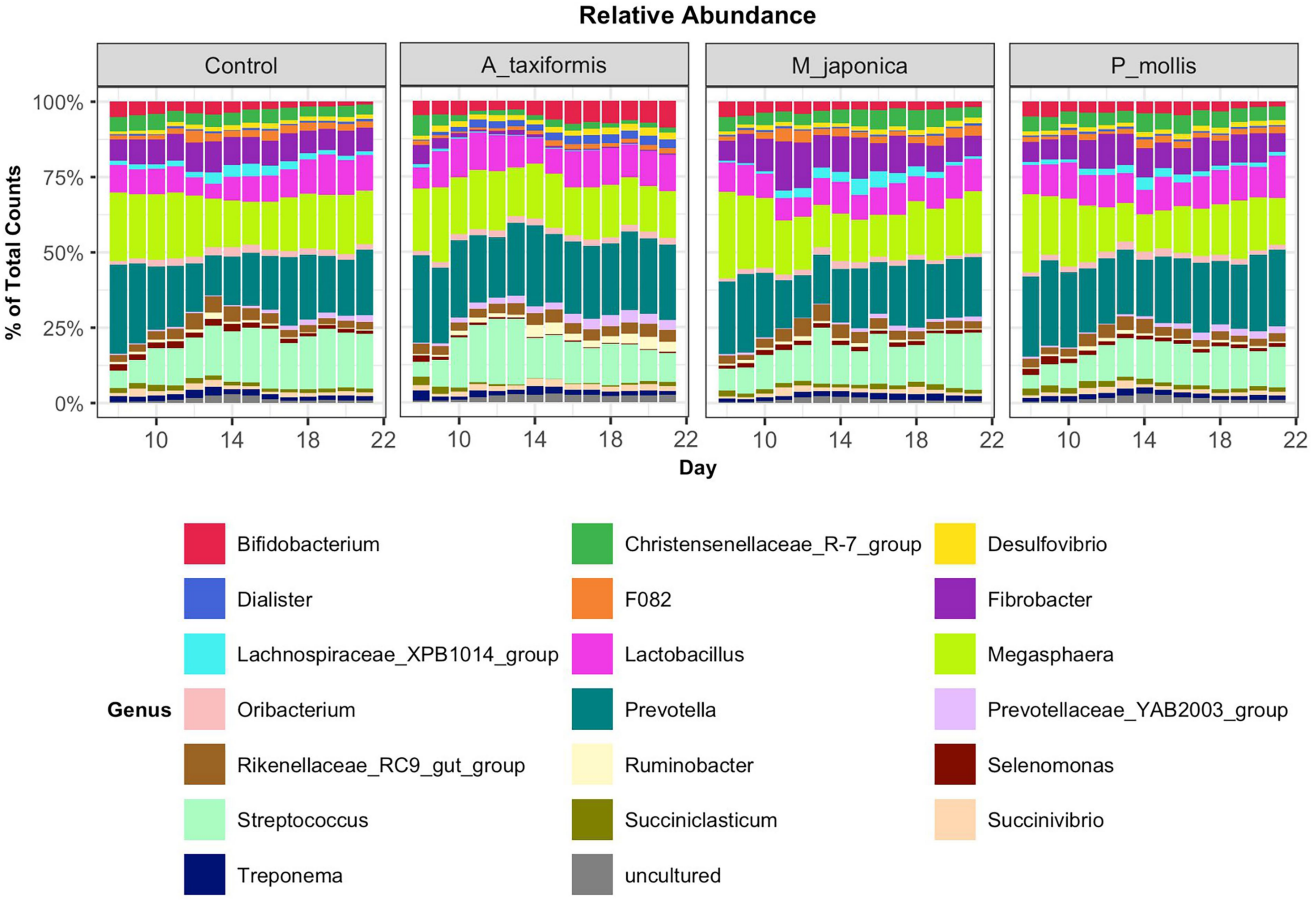
Relative abundances of the 20 most abundant genus-level features across all treatment groups. Abundances were scaled to 1 for ease of presentation.

**Figure 5:**
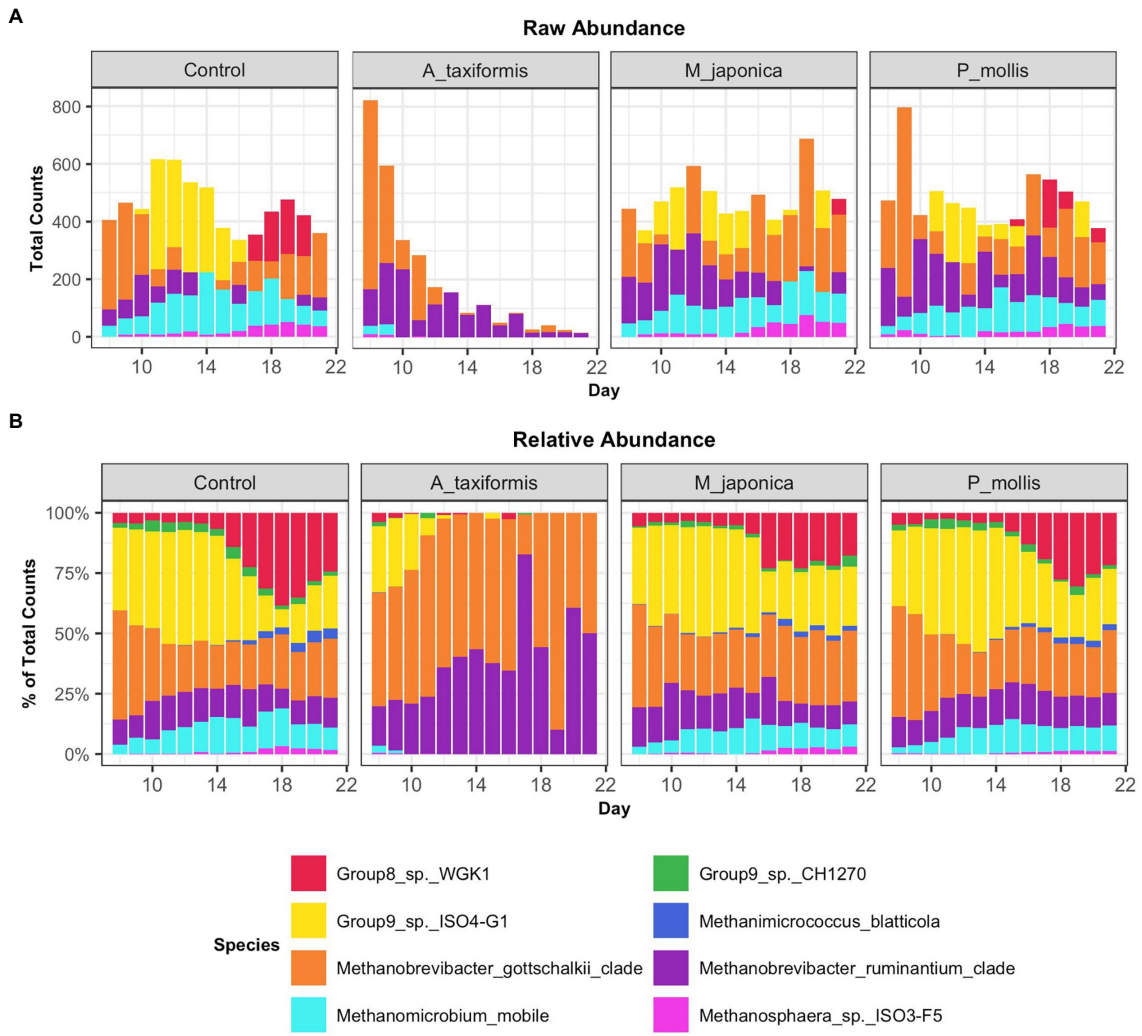
Raw **(A)** and relative **(B)** abundances of methanogenic species across all treatment groups. Values are the daily median across all samples.

Following the removal of sparse ASVs, 1 archaeal and 16 bacterial phyla were identified across all samples. Irrespective of treatment or phase, the microbiomes were dominated by Bacteroidota and Firmicutes throughout, with Proteobacteria and Actinobacteria also present at high proportions. The mean relative abundances for all genera identified in the adaptation, intermediate and stable phase are provided in Supplementary Table S4. The most abundant rumen bacteria included *Prevotella*, *Streptococcus*, *Megasphaera*, *Lactobacillus*, and *Fibrobacter* (Figure 4). The “core” bacteriome was calculated across control and *A. taxiformis* samples and consisted of 32 genera drawn from 8 phyla. The divergence in the core microbial groups between control and *A. taxiformis* samples increased across phases (Supplementary Figure S2) indicating that at least some of these taxa were influenced by *A. taxiformis* supplementation in the RUSITEC. As expected, major ruminal bacteria including *Fibrobacter, Prevotella, Succinivibrio, Megasphaera*, and *Butyrivibrio* were part of the core microbiome. The methanogen community was dominated by *Methanobrevibacter ruminantium, Methanobrevibacter gottschalkii*, and several poorly characterized species belonging to *Methanomassilicoccaceae* (Figure 5). *Methanomicrobium mobile, Methanimicrococcus blatticola*, and *Methanosphaera* sp. were among the less abundant methanogens in the RUSITEC. When raw read counts were visualized, there was a clear decline in the number of sequences attributed to methanogenic species over time in the *A. taxiformis* samples (Figure 5A). Compared to control samples, there was a 86 and 97% decrease in sequences from methanogens in *A. taxiformis* samples in the intermediate and stable phases, respectively. Raw and relative abundances of all microbial taxa are presented in Supplementary Tables S4–S8.

The microbial response underpinning the dramatic reduction in CH_4_ by *A. taxiformis* reported by Terry et al. (2022) was investigated in more detail *via* correlation analysis and differential abundance testing. To examine if bioactives present in the non-methanogenic seaweeds had an impact on the microbiome, testing was also conducted between the control and *M. japonica* samples. Differentially abundant (DA) features were those identified using both tools (see methods). *M. japonica* did not have a major effect on the bacterial community, with just 2 genera responding to treatment during the adaptation phase; *Oribacterium* was more abundant in the seaweed samples (*p* < 0.05), while *Pirellulaceae P1088_a5_gut_group* was more abundant in the controls (*p* < 0.05).

**Figure 6:**
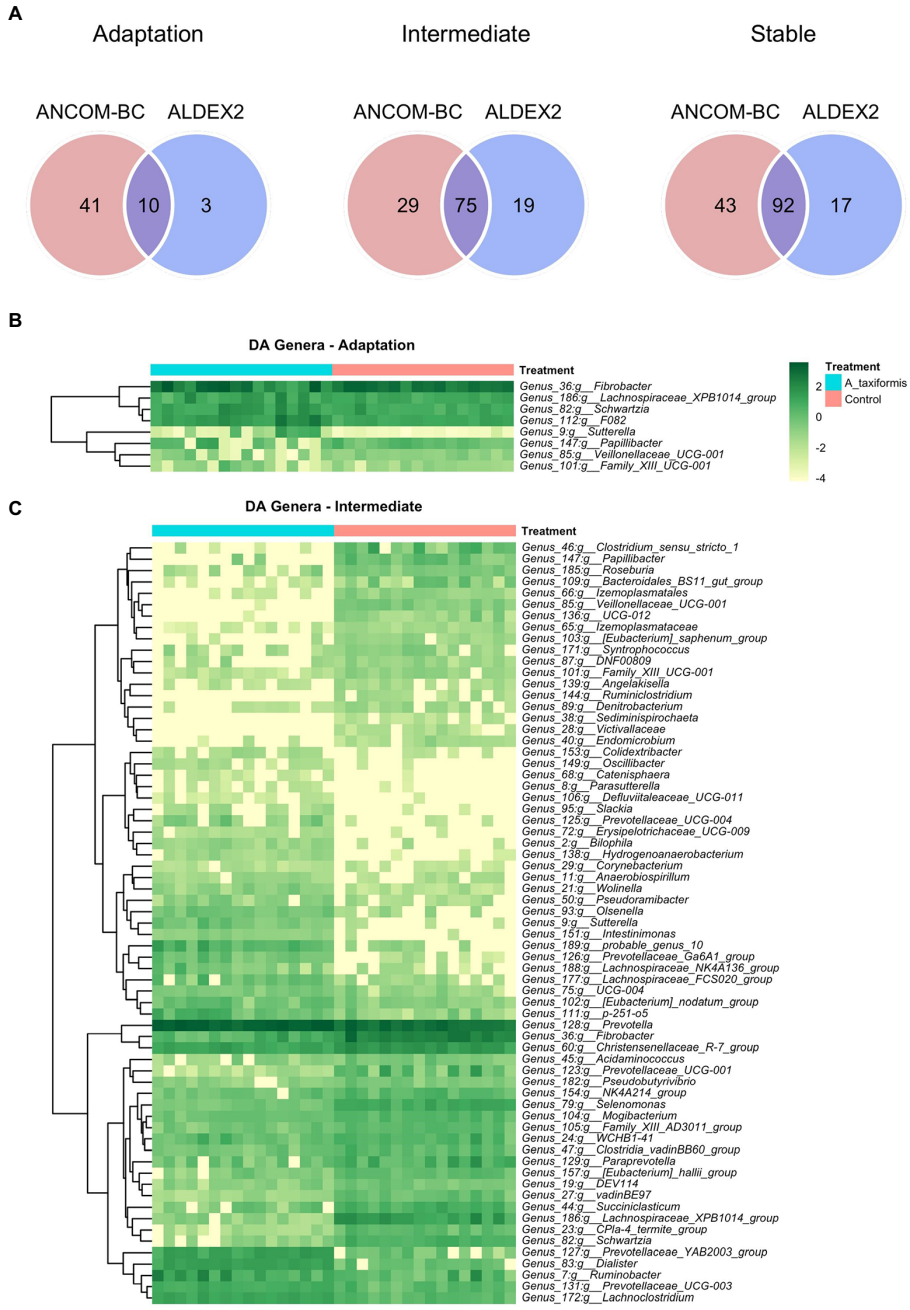
Differential abundance (DA) analysis of bacterial genera. **(A)** Venn diagrams showing the overlap between ANCOM-BC and Aldex2 results. Heatmaps of DA genera for the **(B)** adaptation and **(C)** intermediate phases are shown. Raw count data was log transformed for plotting. Genera denoted as uncultured or unknown are excluded from the heatmaps.

**Figure 7:**
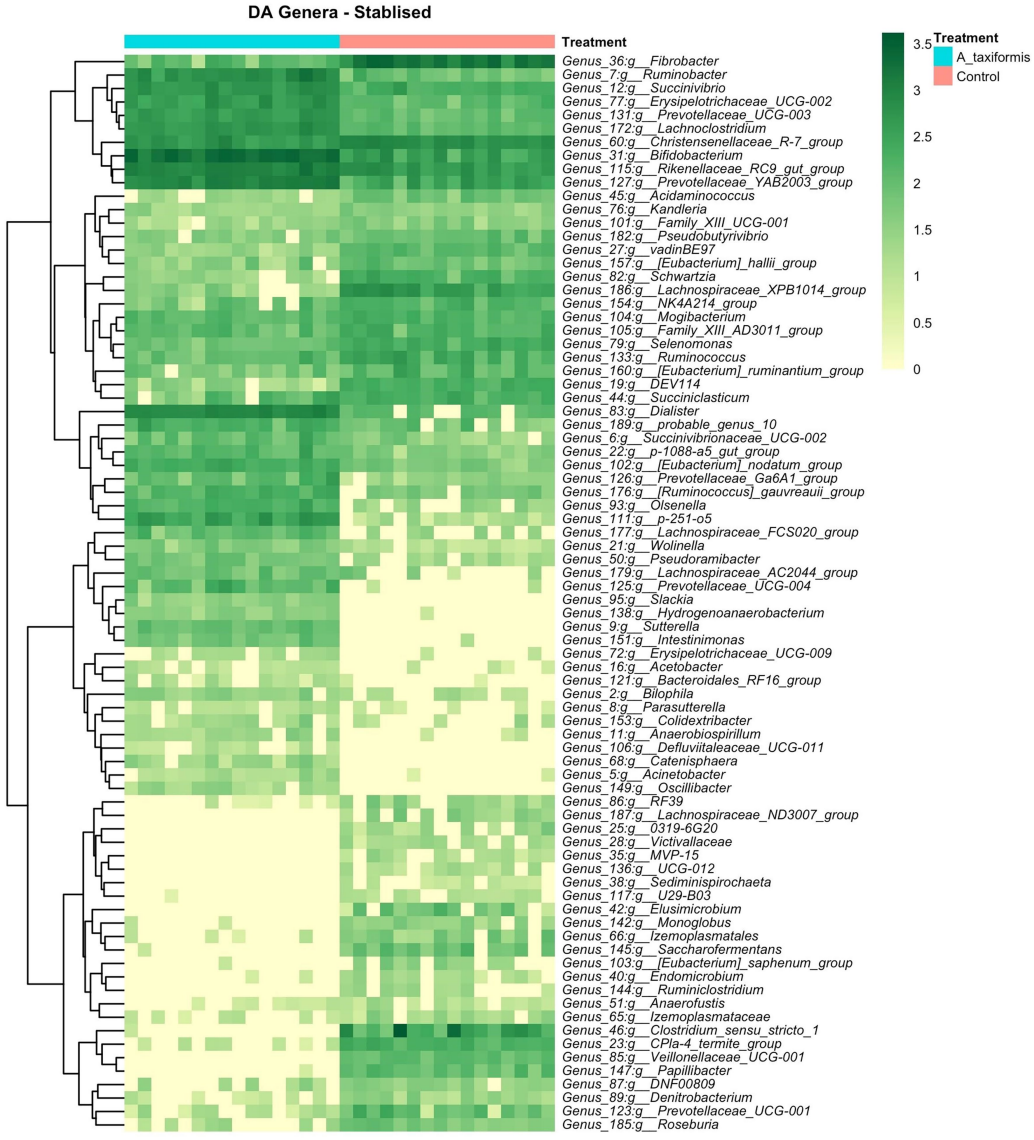
Differential abundance (DA) analysis of bacterial genera. Heatmaps of DA genera for the stabilized phase. Raw count data was log transformed for plotting. Genera denoted as uncultured or unknown are excluded.

The bacterial community exhibited a progressive response to *A. taxiformis*, with 10, 76, and 92 DA bacterial genera identified during the adaptation, intermediate, and stable phases, respectively (*p* < 0.05). ANCOM-BC identified more DA genera throughout the experiment than Aldex2 (Figure 6A). Eight genus-level features were affected by *A. taxiformis* during all 3 phases. Among them, the abundance of *Fibrobacter* was 2.8 fold lower in *A. taxiformis* samples compared to controls in the adaptation phase and 19.5 fold lower in the stable phase (*p* < 0.05). The abundance of *Schwartzia,* was reduced by *A. taxiformis* throughout the experiment by 2 fold in the adaptation phase, 10.8 fold in the intermediate phase, and 17.8 fold in the stable phase compared to the control samples (*p* < 0.05). Reads from *Papillibacter* were no longer detected in the stable phase in *A. taxiformis* samples. *Sutterella* was the only genus with an abundance that was consistently higher in *A. taxiformis* samples (0.05–0.27%) compared to the controls throughout the experiment (*p* < 0.05) whereas, reads assigned to this genus comprised less than 0.01% of the total abundance in control samples (Figures 6B,C, 7). There was a large degree of overlap between the DA taxa in the intermediate and stable phases, with 54 showing the same response to seaweed supplementation in both phases (Figures 6C, 7). Many major rumen bacteria and members of the core microbiome were influenced by *A. taxiformis* in the intermediate and stable phases of the experiment, including increased abundance of *Prevotella*, *Dialister*, *Succinivibrio* and *Ruminobacter* (*p* < 0.05). Members of *Clostridium sensu stricto 1, Roseburia,* and *Ruminiclostridium* were among the genera more abundant in the control samples (*p* < 0.05) in the intermediate and stable phases. Moreover, several minor members of the assemblage responded to *A. taxiformis* in the intermediate and stable phases, including lowered abundances of *Endomicrobium, Denitrobacterium, and Angelakisella* (*p* < 0.05). There were also shifts in the abundances of many unclassified and/or poorly annotated genera belonging to *Prevotellaceae* and *Lachnospiraceae* (*p* < 0.05), as well as other families (Supplementary Table S4).

**Figure 8:**
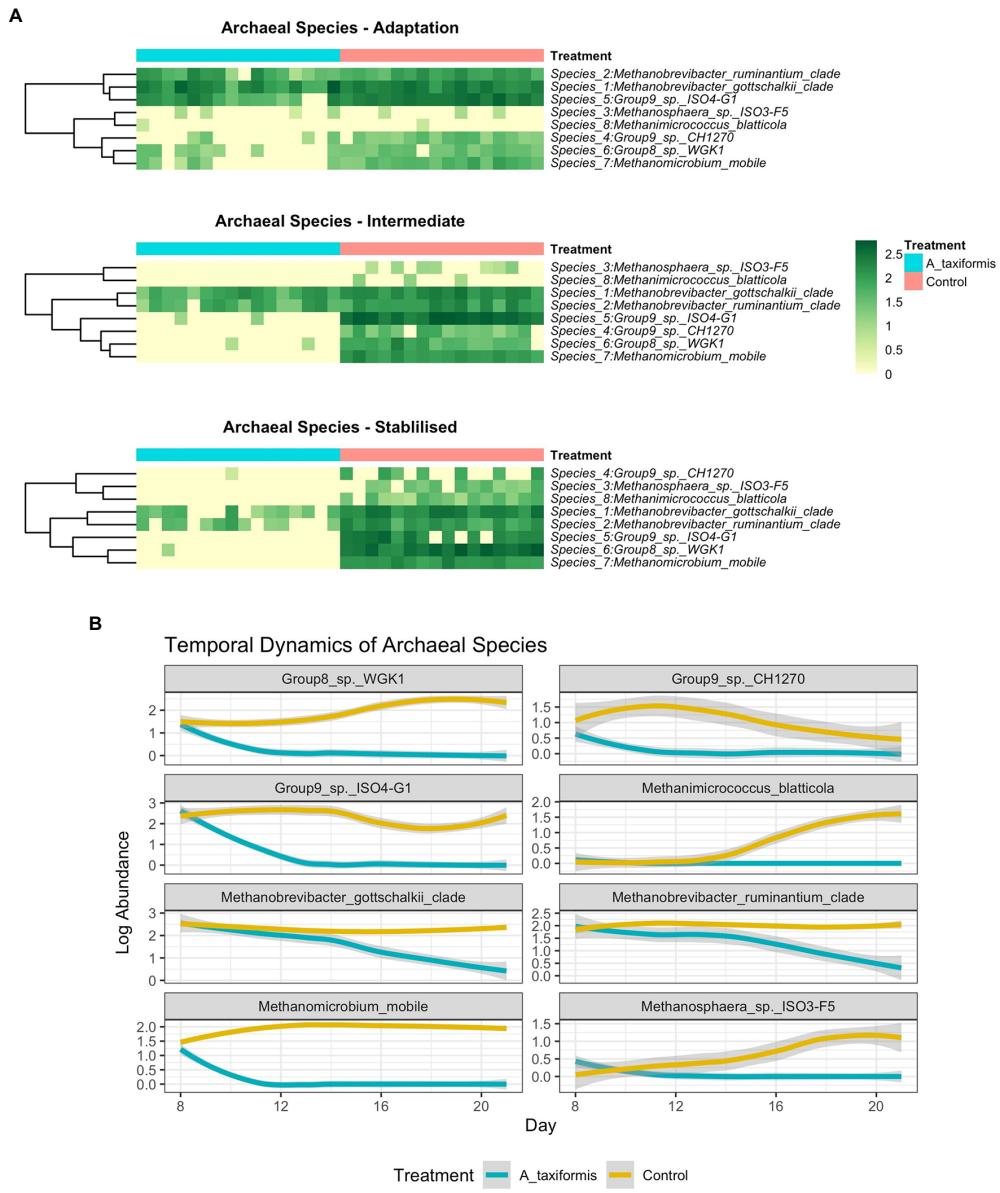
**(A)** Heatmaps depicting archaeal community composition in each experimental phase and **(B)** line plots showing temporal species dynamics throughout the experiment. Raw abundances were log-transformed prior to plotting.

When low-prevalence features were removed, there were 8 archaeal species-level taxa left in our dataset. No archaeal species exhibited a statistically significant response to *M. japonica* supplementation throughout the experiment (*p* > 0.05). The number of archaeal reads recovered from the *A. taxiformis* samples declined drastically in the latter stages of the experiment and sequences from many of the archaea present at the start of the experiment were not detected in *A. taxiformis* samples during the stable phase (Figure 5A). Interestingly, rumen methanogens appeared to show different levels of sensitivity to *A. taxiformis*, with the abundances of *M. mobile* and *Methanomassilicoccaceae* species declining almost immediately, while *Methanobrevibacter* species took several days to drop significantly compared to control samples (Figure 8B). Reads from *M. gottschalkii* and *M. ruminantium* were 2.4 and 2.3 fold lower (respectively) in *A. taxiformis* samples compared to levels observed in the control in the intermediate phase (*p* < 0.05) (Figure 8). In the stable phase of the experiment *M. gottschalkii* and *M. ruminantium* reads were 15 and 7.8 fold lower (respectively) in *A. taxiformis* samples compared to controls. No reads from *Methanosphaera* sp. ISO3-F5, *M. mobile*, *M. blatticola* were identified in *A. taxiformis* samples in intermediate phase samples. A total of 50 reads from *Methanomassilicoccaceae* groups were found in *A. taxifor*mis samples in the intermediate phase. In contrast, reads from these taxa had increased by 1.5 fold in control samples compared to levels found at the start of the experiment. Only reads from *M. ruminantium, M. gottschalkii*, and *Methanomassilicoccaceae* Group 9 were detected in the stable phase samples and reads from these groups were reduced by 93, 87, and 99% compared to the control samples, respectively. The collapse in the methanogen community in *A. taxiformis* samples resulted in a highly sparse data set and complicated the application of widely used tools for identifying differentially abundant microbial taxa. In spite of this challenge, the 97.1% decrease in reads from methanogens in *A. taxiformis* samples compared to the control clearly demonstrates the inhibitory activity this seaweed has on rumen methanogens.

#### 3.3.3. Correlation between microbial communities and fermentation variables

**Figure 9:**
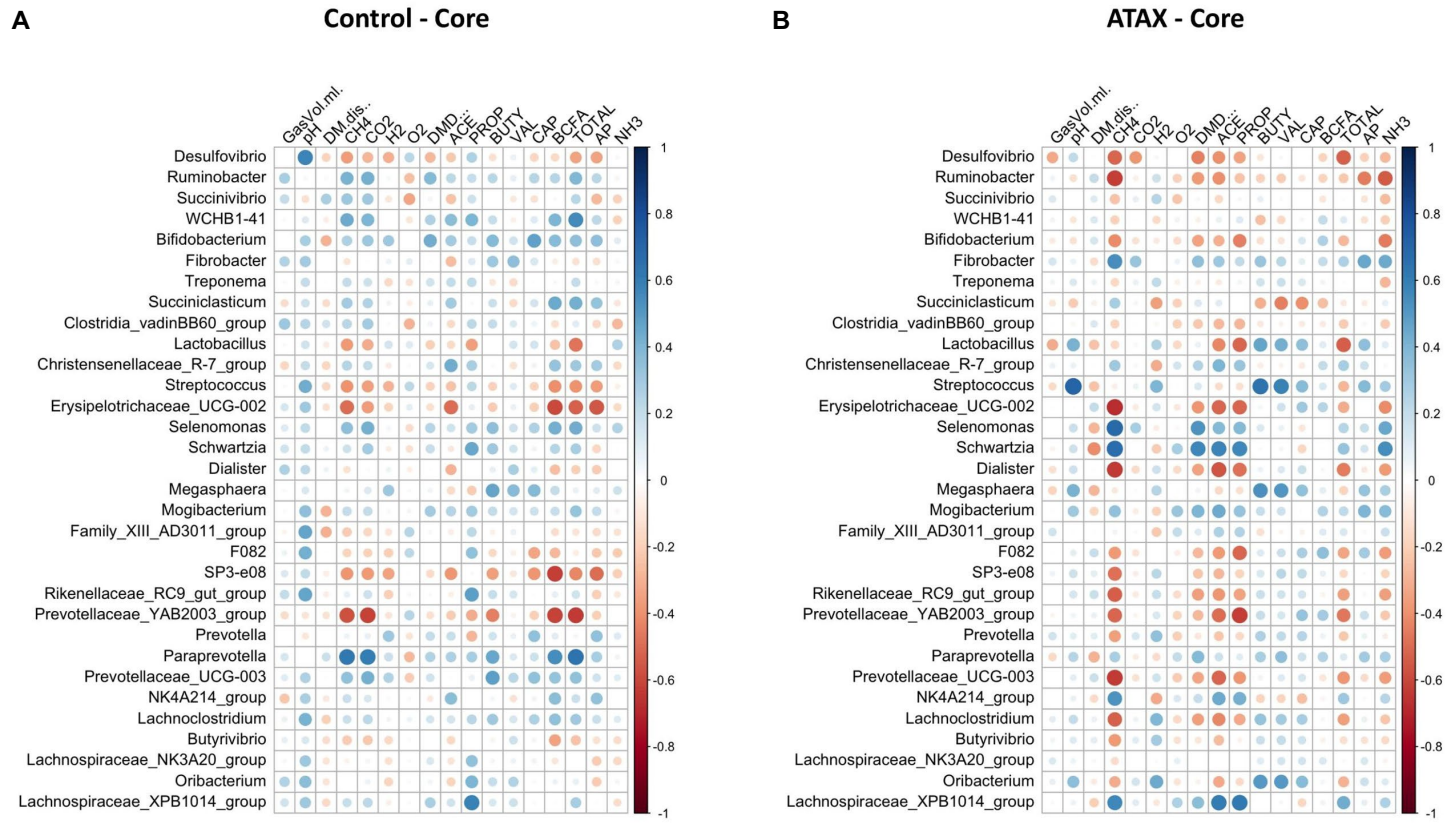
Spearman correlation analysis plots between the core bacteriome of **(A)** control samples and **(B)** *A taxiformis* samples and fermentation variables. Raw bacterial abundances were log-transformed prior to correlation. Only data from control and *A taxiformis* reactors was analyzed. GasVol.ml, total gas volume in mL; DM.dis., dry matter disappearance; CH4, methane; CO2, carbon dioxide; H2, hydrogen; O2, oxygen; DMD, dry matter digestibility; ACE, acetate; PROP, propionate; BUTY, butyrate; VAL, valerate; CAP, caproate; BCFA, branched-chain fatty acids; TOTAL, total VFA; AP, acetate: propionate ratio; NH3, ammonia. Individual sample values for all measurements are provided in the Supplementary material.

Spearman correlation coefficients were used to determine the relationships between fermentation variables reported by Terry et al. (2022) and microbiome features. A relationship was considered strong at an absolute *R* value >0.5 and an adjusted *p*-value <0.05. The core microbes exhibited stronger relationships with fermentation variables in the *A. taxiformis* samples than in the controls. CH_4_ concentration was strongly associated with 13 of the 32 core genera in the *A. taxiformis* samples; *Fibrobacter, Selenomonas, Schwartzia, NK4A214* (*Ruminococcaceae*), and *Lachnospiraceae XPB1014* were all positively correlated with CH_4_ concentration (*p* < 0.05; Figure 9), while *Desulfovibrio*, *Ruminobacter*, *Erysipelotrichaceae* UCG-002 and *Dialister* were among those that exhibited negative relationships with CH_4_ (*p* < 0.05). Conversely, only 2 genera correlated with CH_4_ in the control samples, with *Prevotellaceae YAB2003* exhibiting a negative relationship and *Paraprevotella* a positive one (*p* < 0.05). Strong relationships between core taxon abundances and the molar proportions of individual VFA were also more evident in *A. taxiformis* than controls. *Schwartzia* and *Lachnospiraceae XPB1014* were positively correlated with acetate and propionate (*p* < 0.05), while *Erysipelotrichaceae UCG-002* was negatively correlated with these VFAs (*p* < 0.05). The molar proportion of propionate was also negatively correlated with the abundances of *Lactobacillus, Prevotellaceae YAB3003 group*, and *F082* (*p* < 0.05), while *Dialister* and *Prevotellaceae UCG-003* had a negative relationship with acetate proportion (*p* < 0.05). Butyrate was positively associated with *Streptococcus, Megasphaera* and *Oribacterium* in the *A. taxiformis* samples (*p* < 0.05). Total VFA concentration was also negatively correlated with *Desulfovibrio* and *Lactobacillus* in the *A. taxiformis* samples (*p* < 0.05). The molar proportion of propionate was positively correlated with *Lachnospiraceae XPB1014* in the control samples (*p* < 0.05). Total VFA level was positively correlated with *WCHB1-41* and *Paraprevotella* in the controls (*p* < 0.05), while *Erysipelotrichaceae UCG-002* and *Prevotellaceae YAB2003* group exhibited the opposite relationship. Correlation coefficients and *p*-values for the core genera are provided in the Supplementary Table S9 and presented graphically in Figure 9.

**Figure 10:**
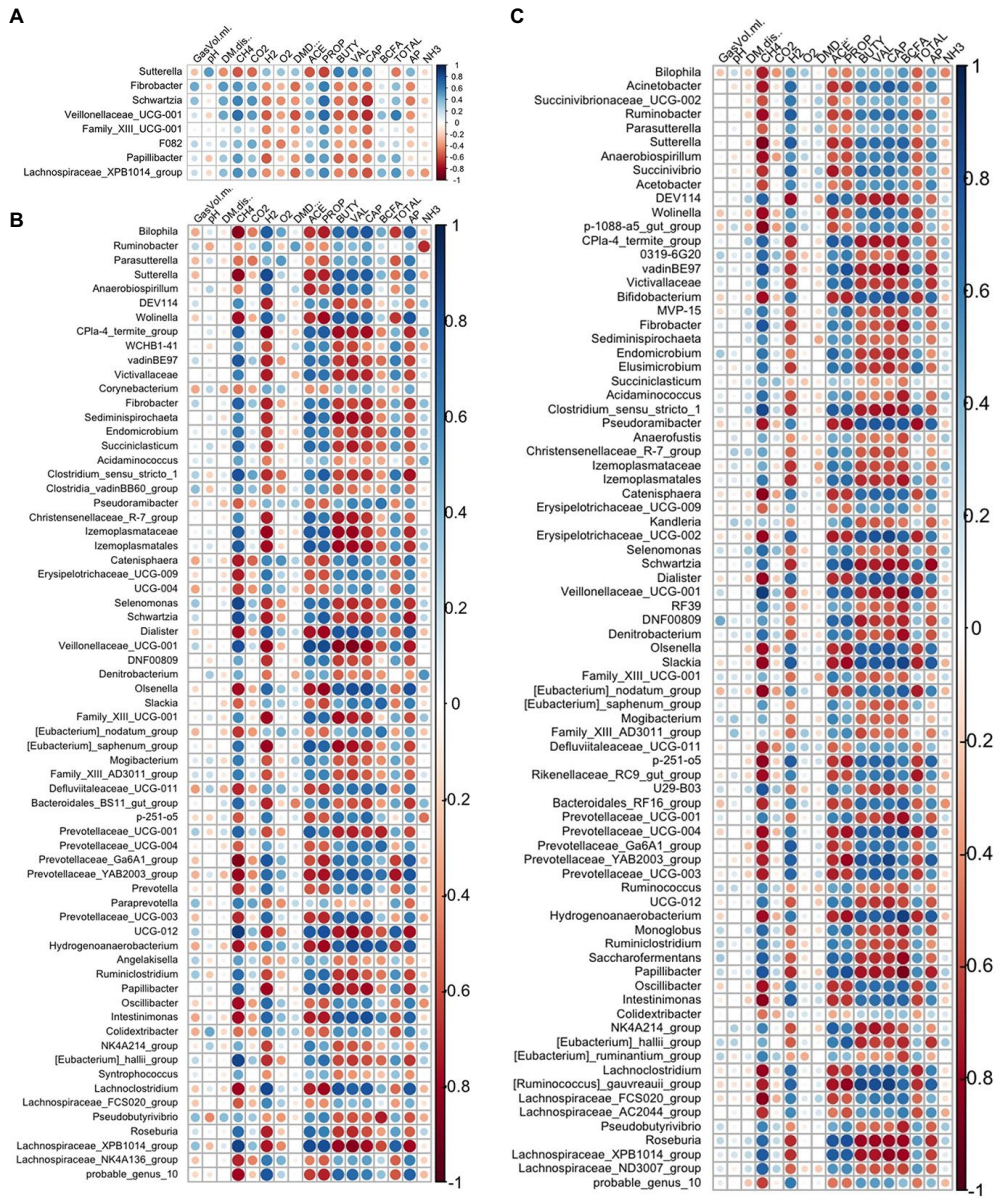
Spearman correlation analysis plots between the differentially abundant genera and fermentation variables during the **(A)** adaptation, **(B)** intermediate, and **(C)** stabilized phases. Raw bacterial abundances were log-transformed prior to correlation. GasVol.ml, total gas volume in mL; DM.dis., dry matter disappearance; CH4, methane; CO2, carbon dioxide; H2, hydrogen; O2, oxygen; DMD, dry matter digestibility; ACE, acetate; PROP, propionate; BUTY, butyrate; VAL, valerate; CAP, caproate; BCFA, branched-chain fatty acids; TOTAL, total VFA; AP, acetate: propionate ratio; NH3, ammonia. Individual sample values for all measurements are provided in the Supplementary material.

Strong relationships were evident between the DA genera (*A. taxiformis* vs. control) and fermentation variables throughout the experiment and largely reflected the taxa identified as differentially abundant between these treatments (Figure 10). In the adaptation phase, CH_4_ and propionate were positively correlated with multiple DA genera including *Fibrobacter, Schwartzia, Veillonellaceae* UCG-001 and *Lachnospiraceae XPB1014 group* (*p* < 0.05), while *Sutterella* had inverse relationships with both (*p* < 0.05). Conversely, caproate was negatively correlated with 5 of the DA genera, and positively correlated only with *Sutterella* (*p* < 0.05) (Figure 10A). In the latter phases of the experiment, almost all the differentially abundant taxa were significantly correlated with CH_4_ concentration and the molar proportions of VFAs (*p* < 0.05) (Figures 10B,C). Total VFA, H_2_ concentration, and the acetate:propionate ratio also exhibited strong relationships with DA taxa in the intermediate and adaptation phases (*p* < 0.05). All correlation coefficients and *p*-values are presented in Supplementary Table S10.

## 4. Discussion

The effectiveness of the red seaweed *Asparagopsis taxiformis* in suppressing enteric methanogenesis in ruminants has been demonstrated both *in vitro* and *in vivo* (Kinley et al., 2016; Machado et al., 2016a; Roque et al., 2019, 2021). Our recent study examining the effects of three red seaweeds, *A*. *taxiformis*, *M. japonica*, and *P. mollis,* on *in vitro* rumen fermentation and gas production confirmed previous observations of the potency of *A. taxiformis* in reducing methanogenesis, with CH_4_ concentrations declining by 95.1% compared to the control treatment (Terry et al., 2022). This mitigation effect was accompanied by reductions in fiber degradation and VFA production which could have negative impacts on animal performance. In contrast, there was no measurable impact of either *M. japonica* or *P. mollis* on methanogenesis or microbial fermentation. There is limited data concerning the microbial response to seaweed supplementation in ruminants. This study presented an opportunity to examine the impact of seaweed supplementation over an extended period on rumen microbes, and to assess if the rumen microbiome shows any evidence of adaptation to seaweeds which might lessen their anti-methanogenic effects over time as has been previously documented (Knight et al., 2011).

The near-total collapse of the methanogen community in the *A. taxiformis* samples (Figures 1A, 5A) was striking and cannot be attributed to temporal shifts in community composition commonly associated with RUSITEC fermenter apparatus (Mateos et al., 2017) or a decline in protozoa associated methanogens (Roque et al., 2019), as the archaeome of the control samples remained relatively stable throughout. We observed a 97.1% reduction in the number of reads from methanogenic archaea in *A. taxiformis* samples compared to controls during of the stable phase of the experiment. There was no suggestion of niche transition among the methanogen species following *A. taxiformis* addition, with the abundances of all major archaea declining throughout the experiment. CCA analysis suggested a strong relationship between the CH_4_ and H_2_ concentrations reported previously (Terry et al., 2022) and methanogen community dynamics. The extent of the decline in methanogen abundance observed here has not, to our knowledge, been previously documented. A recent *in vitro* study supplemented *A. taxiformis* at 5% (w/v) and achieved a similar reduction in CH_4_ production to that observed in this work; however, while *A. taxiformis* decreased the abundance of methanogenic groups over a 96 h period, the reduction was not as dramatic as we observed (Roque et al., 2019). The concentration of bromoform in the supplement used by Roque et al. (2019) was not reported so it is unclear how this compares to the level that is present in the *A. taxiformis* used in our experiment. Our results show that *Methanomassilicoccaceae* species declined in abundance almost immediately (within 24 h) following *A. taxiformis* addition in our study, while it took several days before the *Methanobrevibacter* species were inhibited (Figure 8B). This suggests greater resilience of *Methanobrevibacter* spp. to the seaweed-induced changes in the fermenter microenvironment compared to *Methanomassilicoccaceae,* and would explain the comparatively modest reductions in methanogen abundance reported previously over shorter experimental periods (Roque et al., 2019). *Methanomassilicoccaceae* spp. (formerly Rumen Cluster C) produce CH_4_ *via* the reduction of methyl groups (Poulsen et al., 2013; Lang et al., 2015) rather than *via* the hydrogenotrophic pathway employed by *Methanobrevibacter gottschalkii* and *ruminantium* clades, indicating that this pathway is inhibited to a greater extent by bioactives found in *A. taxiformis*. The disparity in response to seaweed supplementation among methanogen species is likely multifaceted. Bromoform is the principal anti-methanogenic metabolite found in *Asparagopsis* species (Paul et al., 2006) and blocks the transfer of methyl groups as well as serving as an alternative electron accepter (Patra et al., 2017), which perhaps most readily explains the rapid decline of methylotrophic species observed here. Further, changes in bacterial composition would result in shifts in the substrate profile available to methanogens, which could indirectly influence archaeal metabolism to a different extent within the various methanogens found in the rumen. Seaweeds possess a multitude of other secondary metabolites (e.g., phlorotannins) which are known to impact microbial communities (Ku-Vera et al., 2020) and these may have directly or indirectly contributed to methanogen dynamics in the present study. Individual methanogens could also vary in their resistance to inhibitory compounds, and our data may simply reflect a greater resilience of the *Methanobrevibacter* species to the deleterious effects of seaweed bioactives (Ungerfeld et al., 2004). Protozoa-associated methanogens contribute up to 25% of ruminal CH_4_ (Newbold et al., 1995), and numbers of protozoa typically decline over time in the RUSITEC system regardless of treatment (Lengowski et al., 2016; Mateos et al., 2017). While we did not assess the protozoan community in this study, it is likely that a decline in protozoan abundance over time would have at least partially contributed to the reduction in overall methanogen abundance observed here. 16S rRNA data offer limited mechanistic insight, and future studies using shotgun metagenomics or metatranscriptomics may provide clarity as to the mechanisms underpinning these observations.

The reduction in methanogenesis and inhibition of the methanogen community was accompanied by a general depression in microbial activity measured by a decline in VFA production and fiber degradation (Terry et al., 2022). This was mirrored in a substantial change in the bacteriome composition, as evidenced by diversity and differential abundance analyses presented here. We observed strong relationships between core bacterial genera and fermentation variables for the *A. taxiformis* samples. 19 of the 32 core bacterial genera were DA between *A. taxiformis* and control samples during at least one phase of the study. The core microbiome encompasses the most ecologically and functionally important taxa in an environment under given sampling conditions, and treatments that disrupt the core microbiome may have negative implications for the ecosystem as a whole (Neu et al., 2021). The observation that *A. taxiformis* significantly altered majority of the core microbiome members including prominent fiber degraders and VFA producers throughout the experiment suggests that inclusion of *A. taxiformis* in feed could have negative impacts on animal performance.

*Prevotella* is routinely reported as the most abundant rumen microbial genus, prominent in carbohydrate and nitrogen metabolism (Kim et al., 2017). *A. taxiformis* increased the abundance of *Prevotella* 1.6 fold during the intermediate phase of the experiment, while numerical differences in the stable phase did not reach significance. Several poorly characterized genera of *Prevotellaceae* were also increased by *A. taxiformis*. It has been speculated that *Prevotella* spp. may redirect excess H_2_ to propionate when CH_4_ is inhibited in the rumen (Aguilar-Marin et al., 2020). Because propionate levels were reduced by *A. taxiformis* supplementation, the increases in *Prevotella* observed here more likely reflect niche transition of various *Prevotella* species as the abundances of several other established rumen microbes declined.

Acetate, propionate and butyrate are the major VFAs associated with ruminal metabolism (Bergman, 1990). In our companion study, *A. taxiformis* reduced total VFA throughout the experiment and the molar proportions of acetate and propionate in the intermediate and stable phases (Terry et al., 2022). While it is not reliable to directly extrapolate from *in vitro* findings, such changes may have negative impacts on animal performance given the importance of propionate and acetate to gluconeogenesis and fatty acid synthesis in the host, respectively (Bauman et al., 1970; Aschenbach et al., 2010). A major pathway for ruminal propionate production is *via* interactions between succinate-producing and utilizing species (Hobson and Stewart, 1997). *Selenomonas ruminantium* uses succinate in its role as a principal propionate producer in the rumen (Sawanon and Kobayashi, 2006) and A*. taxiformis* reduced the abundance of *Selenomonas* in both the intermediate and stable phases. The abundances of other succinate-utilizers were also significantly reduced, including *Schwartzia* (all phases) and *Succiniclasticum* (intermediate and stable phases) (van Gylswyk, 1995; van Gylswyk et al., 1997). The inhibition of several key succinate-utilizing bacteria could contribute to the reduction in propionate production when *A. taxiformis* is supplemented. Interestingly, *A. taxiformis* increased the abundance of genera containing known succinate producers including *Succinivibrio* (stable phase) and *Ruminobacter* (intermediate and stable phases), suggesting alternative roles for ruminal succinate beyond propionate production during *A. taxiformis* supplementation.

A primary role of the rumen bacteria is the degradation of recalcitrant lignocellulosic biomass by several specialized fiber-degrading bacteria, principally *Fibrobacter succinogenes* and *Ruminococcus* spp. (Ransom-Jones et al., 2012; Dassa et al., 2014). Terry et al. (2022) observed reduced OM and NDF degradation in the *A. taxiformis* samples during the adaptation phase, followed by a recovery of fiber digestion in the stable phase. The reduction in fiber degradation was reflected by the significantly lower abundance of *Fibrobacter*, *Ruminococcus*, and other less common cellulolytic genera like *Ruminiclostridium* in *A. taxiformis* samples compared to the controls (Ren et al., 2019; Karri et al., 2021). The abundance of *Fibrobacter* was also strongly positively correlated with CH_4_ levels and negatively correlated with H_2_ levels in the presence of *A. taxiformis*. Although fiber degradation was only impacted in the adaptation phase, the abundance of *Fibrobacter* was reduced throughout the experiment, and the abundance of *Ruminococcus* was reduced in the stable phase in *A. taxiformis* samples compared to controls. The recovery of OM and NDF digestion suggests that other microbial groups may have filled the niche for fiber degradation in the latter stages of the experiment, although we note that microbial abundance and activity do not necessarily correlate in complex microbial ecosystem (Hunt et al., 2013). Multiple members of the *Lachnospiraceae* family possess cellulolytic capabilities and are capable of butyrate synthesis (Meehan and Beiko, 2014). *Roseburia* is a prominent butyrate producer (Barcenilla et al., 2000) but was inhibited by *A. taxiformis* during the intermediate and stable phases despite butyrate being the only major VFA to increase in molar proportion during supplementation. We speculate that the increased abundance of several poorly described *Lachnospiraceae* genera associated with *A. taxiformis* (e.g., *Lachnospiraceae AC2004, Lachnospiraceae FCS020, Lachnospiraceae NK4A136*) may be responsible for maintaining fiber degradation in spite of the reduced abundance of cellulolytic bacteria and may have contributed to elevated butyrate levels (Terry et al., 2022). *Butyrivibrio* is the predominant butyrate producer in the rumen, and the observation that seaweed supplementation did not have any significant effects on the abundance of this bacterium also supports this hypothesis (Moon et al., 2008).

We also observed significantly higher abundances of *Bifidobacterium* in the *A. taxiformis* samples. *Bifidobacterium* is known for its probiotic properties and can adapt to a wide range of substrates (Pokusaeva et al., 2011). Its role in the rumen of adult cattle is less well defined, but elevated abundance is associated with improved feed efficiency (Abe et al., 1995; McLoughlin et al., 2020), and the increased abundance of *Bifidobacterium* may be an indicator of rumen microbial adaptation to *A. taxiformis*.

Terry et al. (2022) reported no impact of *P. mollis* or *M. japonica* on rumen fermentation profiles, so the observation that neither had a major impact on the microbiome was unsurprising in the context of this study. The anti-methanogenic effect of *Asparagopsis* species is attributed to their high bromoform content (Machado et al., 2018). Chemical analysis of the seaweeds examined in this work indicated that only *A. taxiformis* contained measurable amounts of bromoform, explaining the negligible impact that the other two algae had on methanogenesis. Although *P. mollis* or *M. japonica* did not reduce methane levels, some seaweeds without anti-methanogenic activity have proven to be potent modulators of rumen microbiomes both *in vivo* (Zhou et al., 2018) and in a RUSITEC system (Belanche et al., 2016; Zhou et al., 2018; Künzel et al., 2022) due to the wide range of bioactives present in algae. To our knowledge *M. japonica* has not previously been evaluated for its effect on microbial communities, while *P. mollis* has been found to modify the composition of the gut microbial community in mice (Mendez et al., 2020). While unlikely to have any potential as an anti-methanogenic feed additive, future studies may examine higher doses of *P. mollis* and *M. japonica* for potential prebiotic effects on the rumen and its microbes.

In summary, this study evaluated the impact of three red seaweeds on the bacterial and archaeal communities in a simulated rumen environment over a 13-day period. The red algae that did not have anti-methanogenic activity, *P. mollis* and *M. japonica,* had no measurable impact on the microbiome. In contrast, we found that the inhibition of methanogenesis following *A. taxiformis* supplementation reported in our companion study (Terry et al., 2022) was mirrored by substantial shifts in the microbiome. There was a near-total collapse of the rumen methanogen population following *A. taxiformis* supplementation, with few archaeal reads recovered during the stable phase. Similarly, the suppression in VFA synthesis by *A. taxiformis* was underpinned by inhibition of many taxa involved in acetate and propionate synthesis, and fiber degradation. *A. taxiformis* is receiving enormous attention for its role as an anti-methanogenic feed supplement in ruminants and has recently been commercialized. These data provide the first prolonged exploration of rumen microbial dynamics in response to *A. taxiformis* feeding. In-depth studies using shotgun metagenomics or metatranscriptomics may provide a more comprehensive understanding of ruminal microbial dynamics following seaweed feeding, particularly by allowing for the simultaneous evaluation of all prokaryotic and eukaryotic communities of the rumen.

## Data availability statement

The data presented in the study are deposited in the Short Read Archive (SRA) repository, accession number PRJNA869720.

## Ethics statement

The animal study was reviewed and approved by LeRDC animal care commitee protocol ACC1830.

## Author contributions

TM, DA, KB, RG, and ST designed the experiment. PM, ST, and RG performed the laboratory work. EO’H and RG performed bioinformatics and data analysis. EO’H wrote the manuscript with revisions provided by all authors. RG and KB supervised the experiment and provided resources. All authors contributed to the article and approved the submitted version.

## Funding

Funding was provided by Agriculture and Agri-Food Canada grant ID: J-002363.

## Conflict of interest

The authors declare that the research was conducted in the absence of any commercial or financial relationships that could be construed as a potential conflict of interest.

## Publisher’s note

All claims expressed in this article are solely those of the authors and do not necessarily represent those of their affiliated organizations, or those of the publisher, the editors and the reviewers. Any product that may be evaluated in this article, or claim that may be made by its manufacturer, is not guaranteed or endorsed by the publisher.
