# Supplementary material for: Comparative analysis of macroalgae supplementation on the rumen microbial community: Asparagopsis taxiformis inhibits major ruminal methanogenic, fibrolytic, and volatile fatty acid-producing microbes in vitro
Source: Front Microbiol. 2023 Apr 3;14:1104667. doi: 10.3389/fmicb.2023.1104667 (PMC10109387; doi:10.3389/fmicb.2023.1104667)
Supplement: Supplementary file 1 [file Data_Sheet_1.pdf]

# Supplementation with *Asparagopsis taxiformis* inhibits major methanogenic, fibrolytic, and VFA producing microbes of the rumen *in vitro*

**O'Hara E[1], Moote P[1], Terry, S[1], Beauchemin KA[1], McAllister, TA[1], Abbot DW[1], Gruninger RJ[1*]**

[1]Lethbridge Research Centre, Agriculture and Agri-Food Canada, Lethbridge, AB, Canada.

* **Correspondence:** Dr. Robert J. Gruninger.
Robert.gruninger@agr.gc.ca

**Keywords:** methane, rumen, microbiome, seaweed, livestock, greenhouse gas

## Supplementary Data – Figures and Analysis

### 1. Rarefaction Curves

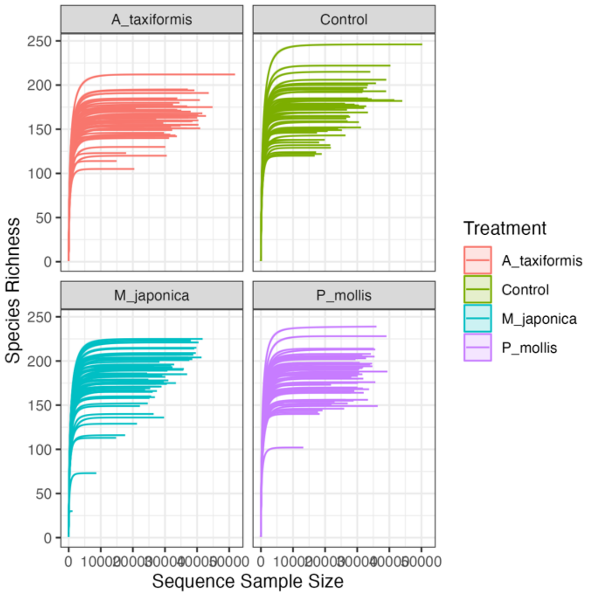


**Figure S1a**: Rarefaction curve of archaeal sequences. Data was randomly subsampled in steps of 50.

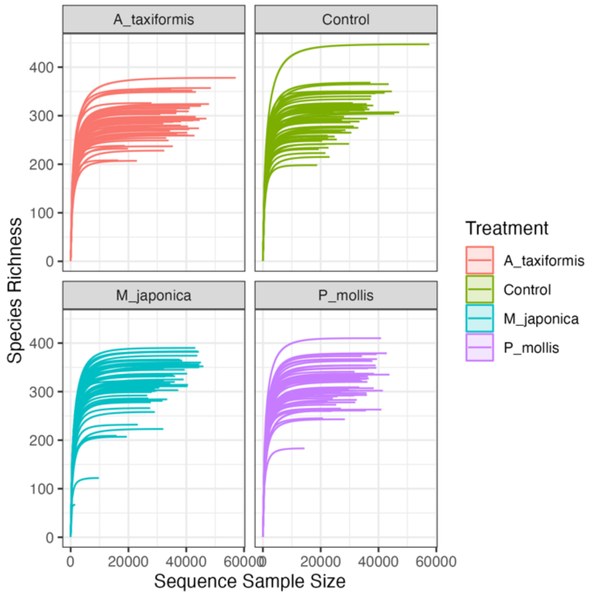


**Figure S1b:** Rarefaction curve of bacterial sequences. Data was randomly subsampled in steps of 500.

## 2. Core Microbiome Heatmaps

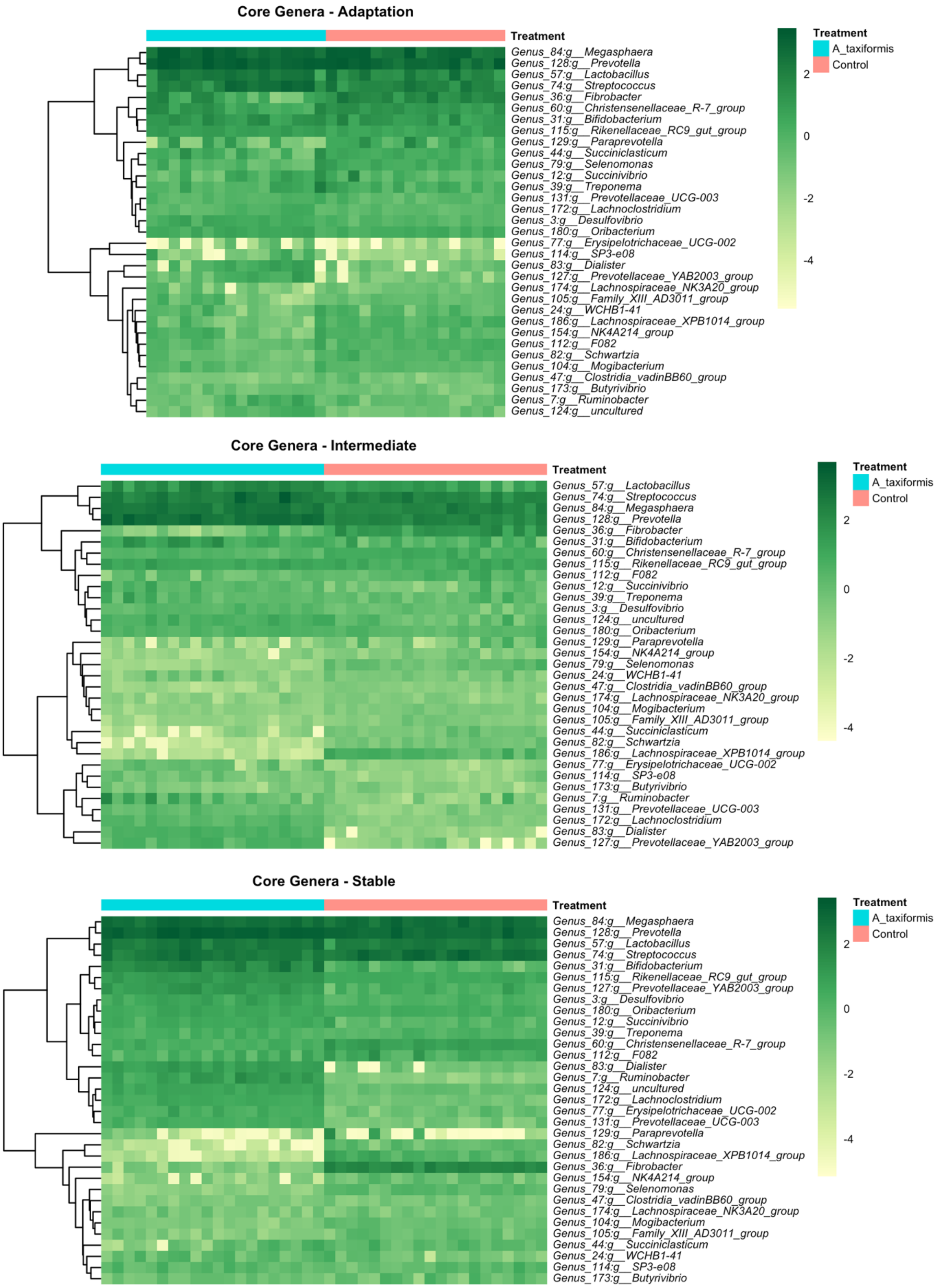


**Figure S2**: Heatmaps depicting the core bacteriome during each phase. Data was log-transformed prior to plotting.

3. **Supplementary Analysis of *Methanocaldococcus villosus* data**

Initial analysis of Q2 data indicated a relatively large proportion of ASVs assigned to *Methanocaldococcus villosus* when taxonomy is assigned using the Rumen and Intestinal Methanogen Database (RIM-DB).

These ASVs were particularly dominant in the *A. taxiformis* samples, where the other methanogens were strongly inhibited. Investigation of their provenance indicated they are native to hydrothermal vents, and thrive at temperatures exceeding 60°C. The RUSITEC system here was maintained at 39°C throughout, casting doubt on the validity of this observation. The confidence of the taxonomic classifications for many of the *M. villosus* ASVs was relatively modest (<0.8), while those of the established rumen methanogens (e.g. *Methanobrevibacter gottschalkii* and *Mbb. ruminantium*) exceeded 99%.

We re-assigned taxonomy to the archaeal reads using several different confidence thresholds – 0.7, 0.75, 0.80, 0.85, and 0.9. The results showed that increasing the assignment confidence threshold to 0.85 totally excluded all *M. villosus* taxa from the dataset, but had little impact on the general structure of the archaeome beyond that. Based on this we proceeded with the data that was assigned at a taxonomic confidence threshold of 0.85.

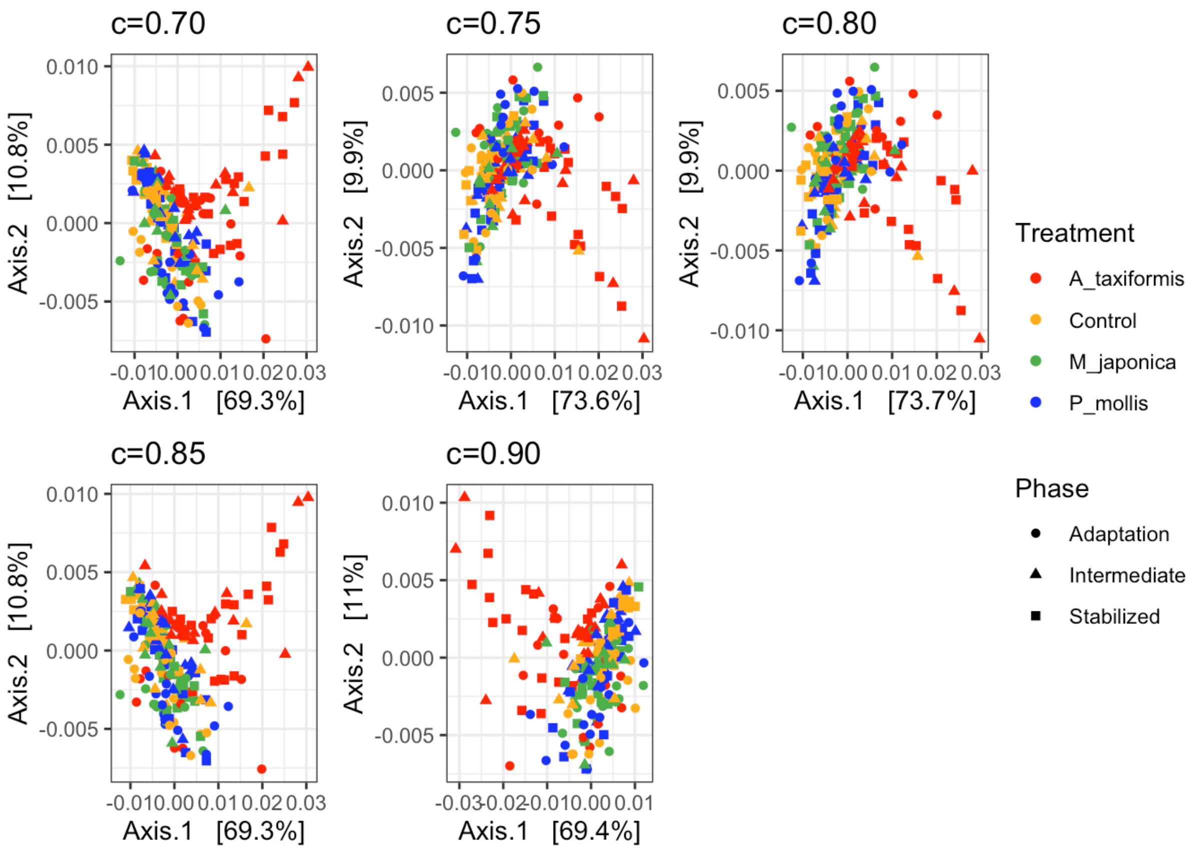


**Figure S3a:** PCoA plots generated from data that was classified at 5 different classification thresholds.

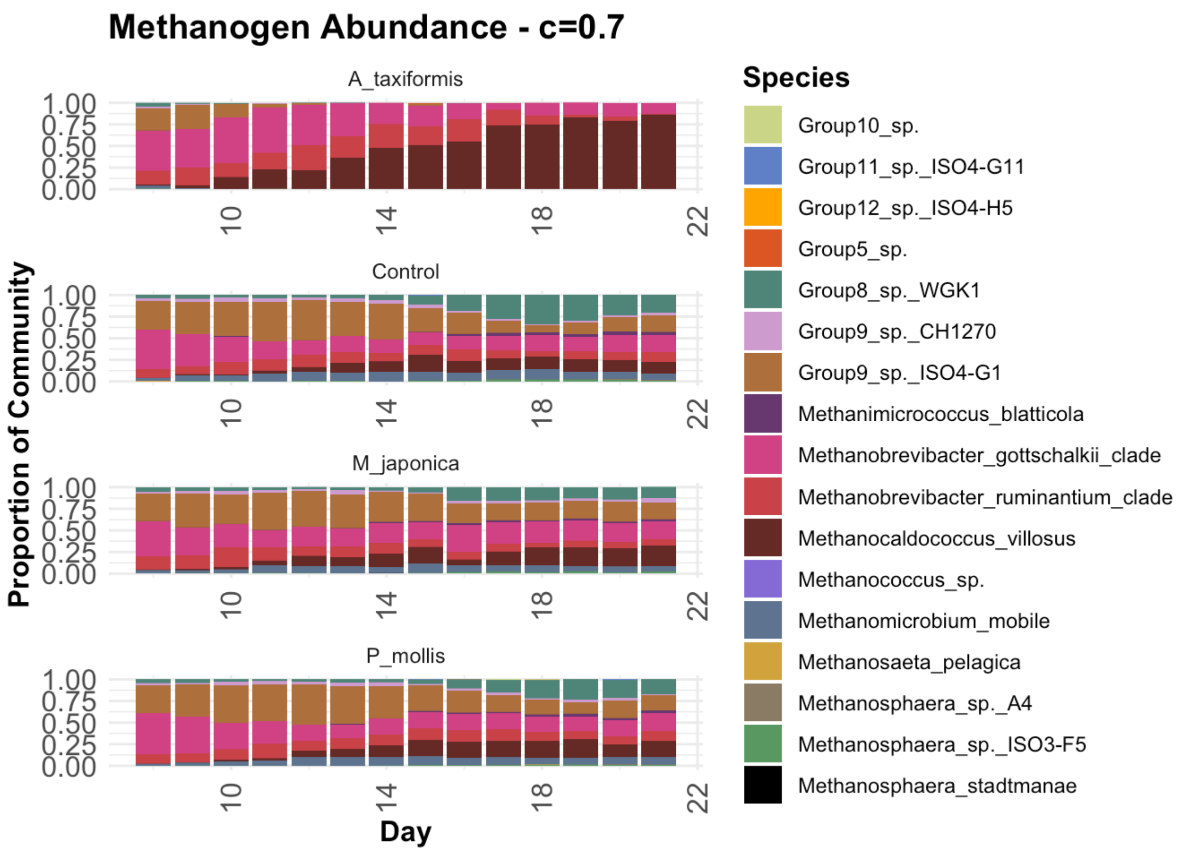


**Figure S3b:** Bar chart depicting the relative proportions of archaeal species following taxonomic classification at a threshold of 0.7

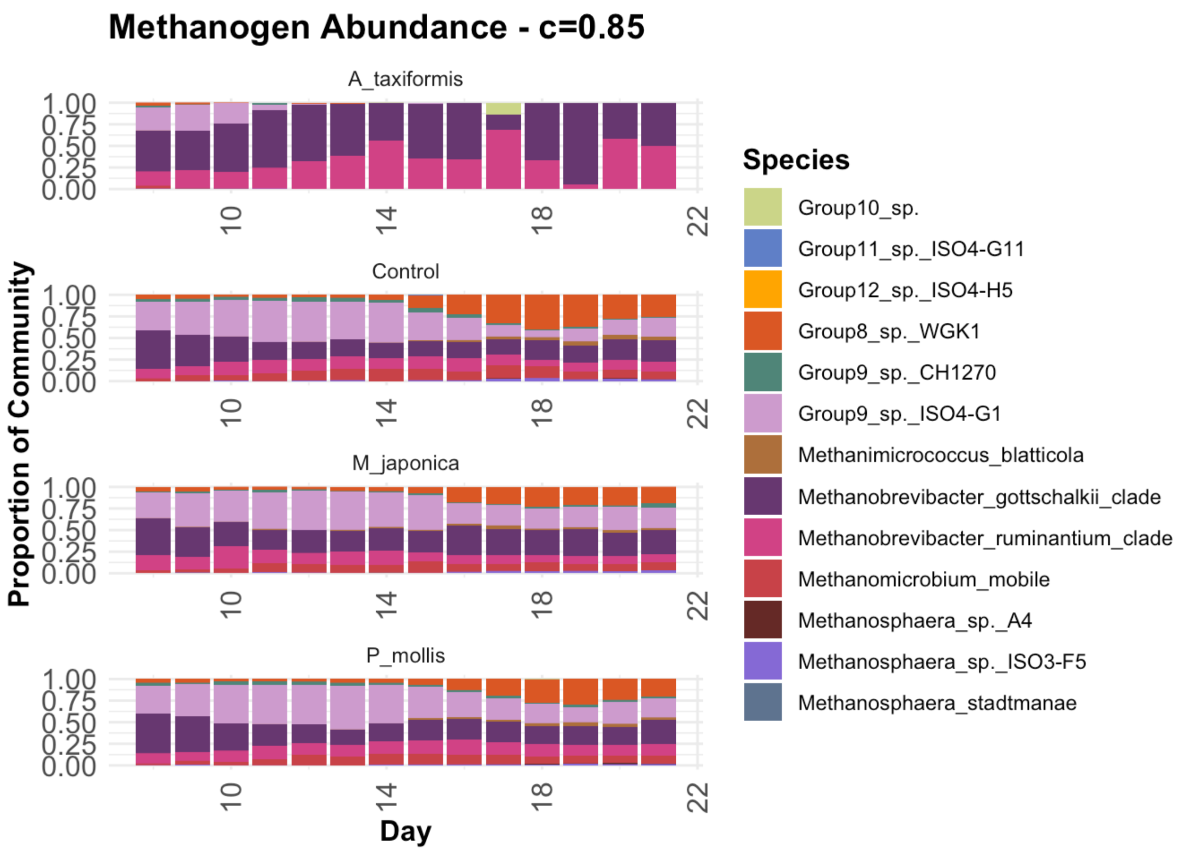


**Figure S3c**: Bar chart depicting the relative proportions of archaeal species following taxonomic classification at a threshold of 0.85
